# Quantifying operational lifetimes for coal power plants under the Paris goals

**DOI:** 10.1038/s41467-019-12618-3

**Published:** 2019-10-18

**Authors:** Ryna Yiyun Cui, Nathan Hultman, Morgan R. Edwards, Linlang He, Arijit Sen, Kavita Surana, Haewon McJeon, Gokul Iyer, Pralit Patel, Sha Yu, Ted Nace, Christine Shearer

**Affiliations:** 1 Center for Global Sustainability, School of Public Policy, University of Maryland, 2101 Van Munching Hall, College Park, MD 20742 USA; 2 Joint Global Change Research Institute, Pacific Northwest National Laboratory, 5825 University Research Court, College Park, MD 20742 USA; 3 Global Energy Monitor, 1254 Utah Street, San Francisco, CA 94110 USA

**Keywords:** Climate-change mitigation, Climate-change policy, Energy policy, Energy infrastructure, Energy modelling

## Abstract

A rapid transition away from unabated coal use is essential to fulfilling the Paris climate goals. However, many countries are actively building and operating coal power plants. Here we use plant-level data to specify alternative trajectories for coal technologies in an integrated assessment model. We then quantify cost-effective retirement pathways for global and country-level coal fleets to limit long-term temperature change. We present our results using a decision-relevant metric: the operational lifetime limit. Even if no new plants are built, the lifetimes of existing units are reduced to approximately 35 years in a well-below 2 °C scenario or 20 years in a 1.5 °C scenario. The risk of continued coal expansion, including the near-term growth permitted in some Nationally Determined Contributions (NDCs), is large. The lifetime limits for both 2 °C and 1.5 °C are reduced by 5 years if plants under construction come online and 10 years if all proposed projects are built.

## Abstract (web summary)

The plant-by-plant retirement needs are not well-understood yet to achieve the rapid transition away from coal use. Here the authors found that operational lifetimes of existing units must be reduced to approximately 35 years to keep warming well below 2 °C or 20 years for 1.5 °C, even if no new capacity comes online.

## Introduction

The Paris Agreement crystallized the world’s commitment to reducing greenhouse gas (GHG) emissions to limit global temperature change to well below 2 °C above pre-industrial levels, while pursuing efforts to limit it to 1.5 °C. Achieving these goals will not be possible without a dramatic reduction in the construction of new coal plants that are not equipped with carbon capture and storage (CCS). Feasible emissions pathways show a steep reduction in the use of unabated coal in global electricity, averaging over 60% reductions in coal emissions by 2030 and approaching zero by 2050^1^. National targets, communicated through the Nationally Determined Contributions (NDCs) to the Paris Agreement, articulate goals for lowering emissions but not explicit plans to move away from coal.

The lack of alignment between current NDCs and the 1.5 °C to 2 °C goals, in terms of both total emissions^2,3^ and sectoral transformations being planned^4–6^, is attracting increasing attention. Regarding fossil fuel infrastructure in particular, studies have evaluated the emissions that would result if current plants live out their historical lifetimes (‘carbon lock-in’ or ‘committed emissions’)^7–14^ and the reserves that must remain in the ground to meet climate policy goals (‘unburnable carbon’)^15^. Other work has highlighted the human health effects of burning coal^16,17^ and the growing financial risks of coal power^18,19^. Scenarios for achieving the Sustainable Development Goals (SDGs) also call for dramatic reductions in coal use^20^. Nevertheless, despite recent activity underscoring the broad-brush need to phase out coal, the specific requirements have not been quantified in the literature or incorporated in many countries’ energy and climate planning processes.

Here we assess what the Paris goals imply for coal power generation at the global level and for several key countries. Our analysis incorporates unit-level data into a global integrated assessment model to analyze the interplay between limits to new coal plants, lifetimes of existing units, and near- and long-term climate goals. Specifically, we ask the following questions: How large are the avoided emissions if proposed coal plants are canceled and existing units retire more rapidly than they have in the past? What reductions in the operational lifetimes of coal plants are compatible with individual countries’ near-term NDCs and the long-term 2 °C or 1.5 °C Paris goals? How would these lifetime limits change if, instead of canceling proposed units, countries continue to bring plants online that are currently in the construction, planning, and permitting stages?

We first build a plant-by-plant dataset of existing and proposed coal units, with global coverage and particular detail for several key countries, using existing public datasets as well as independent data gathering and verification (see Data in Methods). We incorporate this bottom-up data in the Global Change Assessment Model (GCAM, jgcri.github.io/gcam-doc) to specify different coal trajectories and quantify the committed emissions^21,22^ from coal power plants (see GCAM in Methods). Next, we evaluate the gaps between these emissions and the near- and long-term Paris goals, including the gaps for key countries in meeting their NDCs. Finally, we quantify the operational lifetime limits for the global coal fleet to close these gaps if no new plants come online, units under construction are built, or all projects proceed to operation, including those in the planning and permitting stages.

Our analysis includes four scenarios (see Scenarios in Methods). Continued Coal Growth examines a case where proposed plants come online and all plants live out historical lifetimes of approximately 50 years. We compare this scenario to the NDC, well-below 2 °C, and 1.5 °C scenarios. The NDC scenario assumes all individual countries meet their NDC goals—translated into national GHG caps—by 2030. The well-below 2 °C and 1.5 °C scenarios, respectively, limit end-of-century radiative forcing to 2.6 Wm^−2^ and 1.9 Wm^−2^ via a global carbon price on energy-related emissions beginning in 2025. These scenarios are among many in the literature. However, given the scale and speed of the changes required, the general insights emerging from our analysis are robust across scenarios.

We find that, while many countries are still actively planning, authorizing, and constructing new coal power plants, this additional capacity is inconsistent with the long-term Paris goals and even the relatively less stringent near-term NDCs. Cost-effective pathways for meeting the well-below 2 °C or 1.5 °C goals require canceling new coal projects and reducing operational lifetimes of existing units to 35 and 20 years, respectively. The NDCs permit some new plants to be built, but these units add more pressure to accelerate retirement for all units. For example, if all units currently under construction proceed to operation, operational lifetime limits will be reduced by 5 years, to 30 years for a 2 °C goal and 15 years for 1.5 °C.

## Results

### The current state of global coal power

**Fig. 1:**
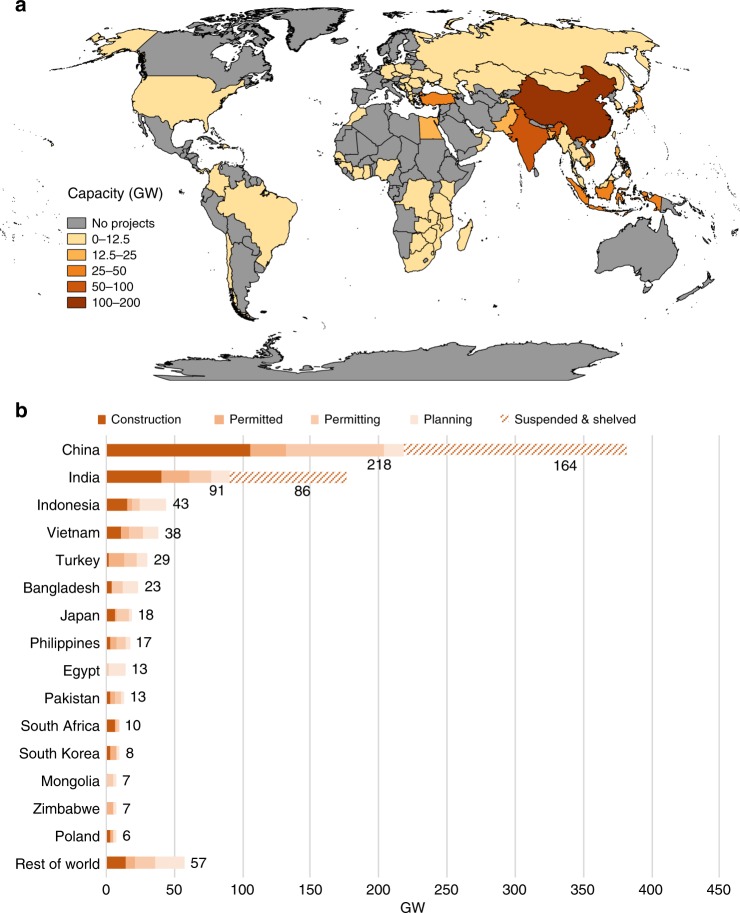
Proposed coal capacity expansion. We find a significant quantity of capacity in planning, permitting, and construction, shown globally (**a**) and by development stage for countries with the largest proposed additions (**b**). Calculations are based on the Global Coal Plant Tracker by Global Energy Monitor (July 2018) and primary, plant-level data collection for China, India, Indonesia, Japan, South Korea, and the US (Source data are provided as a Source Data file)

Despite the stagnation or decline in coal use in the United States (US) and European Union (EU), growth in coal power capacity continues globally and in some places it is rapid. Total installed capacity has more than doubled from 1000 GW globally in 2000 to over 2000 GW in 2015 (ref. ^23^). More than 60 countries are actively planning, permitting, and constructing coal plants (Fig. 1a). We find a total of nearly 600 GW of new capacity is scheduled to come online by 2030, including 223 GW already under construction and 377 GW in the early development phase (Supplementary Fig. 1). Coal plants in the earlier planning and permitting stages are of particular interest because changing course for these units is likely easier than for plants already under construction and can avoid further locking in a carbon-intensive energy system.

Recent regional trends reflect a shift in coal power prioritization from the US and EU to many fast-developing countries in Asia^24^. By 2017, China far exceeded any other country with a cumulative 981 GW installed^25^. The US had the second largest amount of installed capacity at 279 GW^26^, followed by India at 219 GW^27,28^, and the EU at 155 GW^29^. Five countries—China, India, Indonesia, Vietnam, and Turkey—account for about three-quarters of newly proposed coal capacity (Fig. 1b). Even excluding large quantities of suspended and shelved projects, China and India are anticipating large capacity expansions, at 218 GW and 91 GW, respectively. Indonesia is expecting to more than double its current coal operations with 43 GW. Vietnam and Turkey are planning a four- and fivefold increase in coal capacity, with 38 GW and 29 GW, respectively.

### Committed coal emissions and the Paris goals

We incorporate our data on current and proposed coal units into GCAM to quantify the committed emissions^21,22^ from coal power plants and compare these emissions to the near- and long-term Paris goals (see Methods). However, we emphasize that committed emissions are not inevitable. They can be greatly reduced by canceling proposed coal units and retiring existing ones at accelerated rates. We compare these emissions to the modeled coal emissions under of the NDC, well-below 2 °C and 1.5 °C scenarios to estimate the emissions gap, the amount by which committed emissions must be reduced in each year to meet these near- and long-term climate goals cost-effectively.

**Fig. 2:**
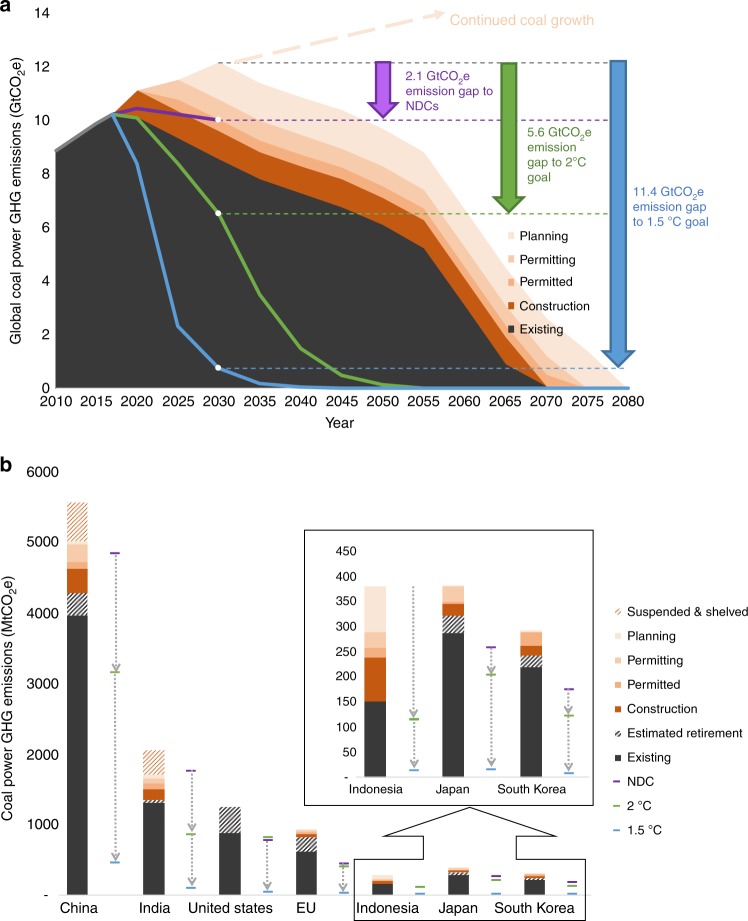
Emissions gaps from coal infrastructure. Emissions from existing and proposed coal plants create gaps for achieving the NDC (purple), well-below 2 °C (green), and 1.5 °C (blue) targets globally (**a**, shown over time) and for key countries and regions (**b**, shown for 2030). Emissions pathways to comply with climate goals are based on country pledges for the NDC scenario and cost-effective global reductions for the well-below 2 °C and 1.5 °C scenarios. Estimated retirements in China, the US, and the EU are based on either unit-level announcements or national targets and policies; estimates in India, Indonesia, Japan, and South Korea are based on a 50-year unit lifetime. (Source data are provided as a Source Data file)

Coal plants, once in place, can run for several decades. Coal projects that are under development today thus have the potential to generate emissions throughout the century, with implications for achieving near- and long-term climate goals. Specifically, completing all proposed projects would lead to 3.6 GtCO_2_e in additional GHG emissions in 2030 if plants operate through the end of the historical average lifetime (~50 years). This quantity is larger than the 2.1 GtCO_2_e emissions gap in the coal power sector for meeting aggregate, economy-wide NDC targets in 2030 (Fig. 2a). Even canceling projects at early permitting and planning stages, where construction has not yet begun, can reduce emissions by roughly the amount of the current gap.

However, the cost-effective emissions gaps in the coal power sector to meet the long-term 2 °C and 1.5 °C goals are greater than those for the NDCs, reaching 5.6 and 11.4 GtCO_2_e, respectively, in 2030 (Fig. 2a). It is widely recognized that the NDCs lack sufficient ambition to achieve the long-term goals of the Paris Agreement^30,31^. We find that even without implementing any new projects, the 2030 emissions from the existing coal fleet far exceed what would be consistent with the long-term Paris goals. To close this gap, canceling all new projects, even including those already under construction, is not enough. Achieving long-term climate goals requires accelerated retirement of existing infrastructure.

The story across key countries and regions is consistent with our global results: the committed emissions from current and proposed coal generation are inconsistent with the near- and long-term Paris goals. Although some NDCs allow more room for new coal plants than others, they will all be difficult to achieve if capacity remains at the levels projected by our unit-level analysis. For some countries (including China and India), achieving the NDC targets requires reducing proposed coal projects. For others (including the US, EU, Japan, and South Korea), it also depends on shutting down existing facilities. More importantly, no country can stay on track with the long-term Paris goals without accelerating coal retirement along with canceling new projects (Fig. 2b).

### Lifetime limits for coal power plants

**Fig. 3:**
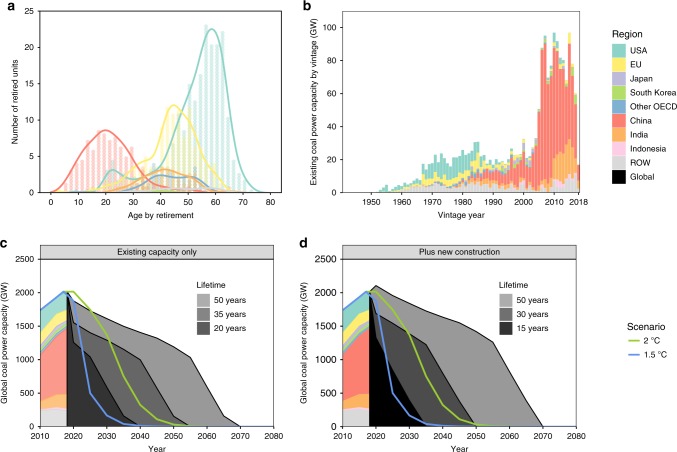
Lifetime limits for coal power plants. We show lifetimes of retired units (**a**) and the vintage year of existing units (**b**) and calculate global coal capacity under different plant lifetimes, compared to capacity levels consistent with a well-below 2 °C (green) and 1.5 °C (blue) pathway, for a case where no new coal plants are built (**c**) and where plants currently under construction come online as scheduled, but those in planning or permitting stages are not built (**d**). Capacity pathways for the well-below 2 °C and 1.5 °C scenarios use a least cost global mitigation scenario starting 2025. Allowing plants currently under construction to come online shortens the retirement timeline by 5 years (i.e., 35- versus 30-year lifetimes for the 2 °C scenario and 20- versus 15-year lifetimes for the 1.5 °C scenario)

Historically, coal plants have retired at an average lifetime of 46 years globally^29^, but in many cases they can operate for 50–60 years or longer^32^ (Fig. 3a). Looking at individual plants, 320 GW or 16% of global installed capacity is expected to shut down by 2030, counting both announced retirements and plants whose lifetimes would exceed 50 years. However, achieving the 2 °C goal requires a 720 GW or 36% reduction in capacity by 2030, and achieving the 1.5 °C goal requires a 1890 GW or 94% reduction (Supplementary Fig. 2). If no new capacity comes online, global coal phase-out can be aligned with the 2 °C goal by reducing operational lifetimes to 35 years; that is, all units retire after 35 years of service, including immediate retirement for plants whose lifetimes exceed this limit (Fig. 3c).

Further reducing operational lifetimes to 20 years aligns with the 1.5 °C goal. These lifetimes are at the lower limits of the historical range in any country (Fig. 3a). Even the current average Chinese plant lifetime of 24 years—reflecting policy pressures to shut down small, dirty units to address local air quality concerns^33^—exceeds this limit. Moreover, bringing new plants online implies further reductions in coal fleet lifetimes. If plants currently under construction are completed as scheduled, the operational lifetime (for both existing and new units) is shortened by 5 years, to about 30 years for the 2 °C goal and about 15 years for the 1.5 °C goal (Fig. 3d). These lifetimes are further reduced by another 5 years if the plants in the planning and permitting stages are also built (Supplementary Fig. 3).

Our analysis applies the same lifetime limits to all units, but it has different implications across countries. Most of the US and EU coal fleets came online in the 1970s and 1980s, whereas the majority of Chinese and Indian capacity was installed more recently, beginning around 2005 in China and 2010 in India (Fig. 3b). Therefore, with the same lifetime limits, aging units in developed countries shut down first, while newer fleets in developing countries can stay online till later. A 35-year limit eliminates 89% and 79% of existing capacity in the US and EU by 2030, respectively, but only 12% and 20% in China and India (Supplementary Fig. 4). However, this headroom is limited for meeting the 1.5 °C goal. A 20-year limit increases US and EU retirements to 95% and 89%, respectively, by 2030 and substantially reduces capacity in China and India to 65% and 43% of current levels (Supplementary Fig. 4). While unit age imperfectly captures the likelihood of retirement, retired plants tend to be older, smaller, less efficient, and highly polluting, making it unprofitable to comply with environmental regulations^34^ or compete with alternative technologies^35^.

## Discussion

Our assessment has uniquely quantified operational lifetime limits of coal plants to cost-effectively meet near- and long-term climate goals, by incorporating plant-level data into a global integrated assessment model. We find that, compared to the historical average lifetime of approximately 50 years, retiring existing units once they reach a 35-year lifetime can limit warming to 2 °C; with a 1.5 °C limit, this lifetime is reduced to 20 years. These lifetime limits are 15 and 30 years shorter, respectively, than the typical average lifetime, an important insight for decision-makers assessing new energy investments. We also find that the NDCs lack sufficient ambition not only in emissions reductions but also by permitting near-term infrastructure investments that are inconsistent with long-term goals. The risk of implementing new projects is large—even allowing plants currently under construction to come online would further reduce lifetime limits for all units by 5 years, and limits are reduced by ten years if projects in earlier development stages also move forward.

As countries continue to pursue coal power for various reasons—including rich domestic resources^36^, growing energy demand^37^, and concerns about grid stability and reliability—the possibility of accelerated retirements and shorter operational lifetimes raises questions on the financial viability of new coal investments. Coal power is increasingly seen as unprofitable and uneconomic: 42% of coal plants currently operate at a loss^18^. This trend will likely continue with the implementation of climate policies and rapid cost reductions in low-carbon technologies^38^. Early retirements could further reduce earnings and compound financial losses. The opportunity costs of these retirements depend on plant location; there is significant variability in lifetimes and payback periods across countries and individual units^18^. Country-level average lifetimes may vary by 15 years in either direction, compared to the global average (Fig. 3a). Historical lifetimes may also not be representative of future values due to changing technical, economic, and political constraints.

There exist diverse views on whether CCS should be part of a deep decarbonization strategy, and we do not take a position in this paper. Some countries may attempt to deploy CCS to extend the use of coal. However, retrofits of existing plants can be expensive^39^, and renewables may be less costly than new or retrofitted CCS plants in many locations^40^. How quickly conventional fossil fuel generation must be phased out also depends in part on the quantity of negative emissions, for example using bioenergy with CCS (BECCS), in the latter half of the century. Our core scenarios constrain the use of bioenergy to a maximum of 200 EJ per year globally. This limits negative emissions from BECCS to approximately −15 GtCO_2_ by 2100. Placing more stringent limits on negative emission technologies (whose large-scale deployment in energy models has been criticized on feasibility and sustainability grounds^41^) will require further accelerating the phase-out of coal. Specifically, reducing the annual bioenergy ceiling from 200 EJ to 150 EJ decreases the lifetime limit by about 5 years (Supplementary Fig. 5), a comparable effect to bringing all plants currently under construction online.

Specific policy efforts that target coal use are critical for avoiding the committed emissions from global coal plants, beyond the pledges made in the current round of NDCs. Recent examples include country- and state-level plans to shut down coal plants though the Powering Past Coal Alliance^42,43^, national limits on coal consumption^44^ and new construction^45^, reductions in multilateral development banks’ financing of coal projects^46^, rising citizen opposition to coal mining^47^ and coal-related air pollution^48^, and public initiatives such as Keep It in the Ground^49^ and Beyond Coal^50^. These developments highlight awareness of the consequences of coal use and suggest that targeting fossil fuel infrastructure may be a politically feasible approach to emissions reductions^51^. Information about the lifetime limits for achieving climate goals can provide further support for these policies.

Developing countries will face additional challenges in the near future, with electricity demand expected to grow and a large amount of new coal infrastructure already in place. Efforts to accelerate coal retirements in those countries can seek to minimize trade-offs with energy access and economic development by pursuing parallel initiatives to deploy low-carbon energy and energy efficiency and address air quality and human health concerns. However, serious political and societal conversations on the energy transition will likely be needed in all countries to underpin a rapid and full coal phase-out^52^, particularly since many older and inefficient coal units in countries like China have already retired^53^. While significant progress has been achieved by widely implementing air pollutant controls^33^, coal power expansion may not be effectively scaled down without direct efforts to cancel new projects and accelerate retirement of existing fleets worldwide^54^.

## Methods

### Data

Our data are based on unit-level assessments of existing and newly proposed coal plants worldwide. Data on existing plants are primarily taken from the Global Coal Plant Tracker by Global Energy Monitor^29^. We cross-check with aggregate data from other sources^25–28^ and modify the data at the national level as needed. In this analysis, we use the year when individual units began operating (Fig. 3b) to estimate retirements based on a fixed lifetime.

For newly proposed coal power plants, we conduct primary, plant-by-plant data collection on six key countries of interest—China, India, Indonesia, Japan, South Korea, and the US. We track the proposed new capacity at different stages of project development. Globally, about 37% of proposed plants have already started construction, more than 16% have been authorized by their government (permitted), about 28% are going through the permitting process, and the remaining 19% are at very early planning stage (Supplementary Fig. 1). We also gather unit-level data on the proposed capacity, combustion technology, local air pollutant control technology, location, developer(s), project utilization, and other characteristics. All information is updated as of September 2018. We identify the proposed projects and gather information from various data sources, including national and local energy development plans, public notices of project permitting processes (such as environmental impact assessments), coal industry status reports, power company websites, and a variety of news channels.

### The Global Change Assessment Model

GCAM (jgcri.github.io/gcam-doc/) is an integrated assessment model that represents and links the world economy, energy, agriculture, land use, water, and climate systems. It is designed to explore interactions between complex systems and gain insights about long-term trends. GCAM has been widely used to produce scenarios for international and national assessments, including the Intergovernmental Panel on Climate Change (IPCC) reports^1,55–57^, the Representative Concentration Pathways (RCPs)^58^, and the Shared Socioeconomic Pathways (SSPs)^59^. We use GCAM 5.0 in this analysis. In this version, the world economy and energy systems operate across 32 geo-political regions, and land allocation and agricultural production are modeled across 235 land and water units.

Specifically, GCAM takes in assumptions about population growth and changes in labor productivity, along with representations of resources, technologies, and policies, and solves for the equilibrium prices and quantities of various energy, agricultural, and GHG markets in each 5-year period from 2010 (the calibration year) to 2100 at different spatial resolutions. Primary energy (i.e., coal and other fossil fuels), agricultural products, and biomass are traded globally. GCAM tracks emissions of 16 GHGs, aerosols, and short-lived species endogenously based on the resulting energy, agriculture, and land systems activity. Emissions are then passed to the climate carbon-cycle module and converted to concentrations, radiative forcing, temperature, and other responses to the climate system^60^.

In this study, we use bottom-up data at the individual unit level to derive alternative pathways for coal technologies in GCAM. We first determine the generation trajectory for each plant in our database. A coal unit built in a year is assumed to continue to operate until the end of an exogenously specified lifetime at a region-specific, constant capacity factor (Supplementary Table 1). We then aggregate unit-level trajectories to obtain coal generation pathways at the level of the GCAM regions and model periods. These trajectories are implemented as constraints on the model’s output and used to quantify the committed emissions (with GCAM efficiency assumptions, Supplementary Table 2). Results are compared against GCAM’s standard Paris Pledge scenarios (see Scenarios below) to calculate the emission gaps in 2030 and identify the lifetime limits that are compatible with the well-below 2 °C and 1.5 °C goals.

### Scenarios

We assess and compare GHG emissions from conventional coal power generation across four scenarios: Continued Coal Growth, NDC, well-below 2 °C, and 1.5 °C. Energy demand trajectories in all four scenarios are based on a middle-of-the-road socioeconomic pathway (SSP2)^61^. However, since coal generation projections and/or temperature targets are implemented in our scenarios, the fuel mix in energy supply, as well as the associated GHG emissions from power generation, may differ from that in the SSP2.

The Continued Coal Growth scenario assumes power generation from conventional coal will follow current national trends, while all other sectors follow the reference scenario without additional emissions reduction efforts. Historical data on coal generation are calibrated up to the year 2015 in GCAM, using country-level data^62^. After 2015, we use unit-level data to simulate existing capacity up to 2018 and proposed capacity that will be deployed up to 2030. Specifically, all capacity currently under construction comes online by 2020, all capacity that is permitted or still in the permitting process comes online by 2025, and all the planned capacity (including the suspended projects in China as well as the shelved projects in India) comes online by 2030. Retirement of existing capacity is estimated based on current plans and policies in China^63^, the US^64^, and the EU^42^, and based on a 50-year power plant lifetime for all other countries. Future coal generation by region is calculated by applying current, region-specific capacity factors (Supplementary Table 1).

The NDC scenario translates all individual country commitments under the Paris Agreement into national emission constraints by 2030. The analysis is based on a scenario developed in Fawcett et al.^3^. This scenario assumes that countries meet their NDC goals—translated into national GHG emission caps—by 2030. After 2030, countries continue to decarbonize their economies at the same annual decarbonization rate (i.e., reduction in carbon intensity per unit gross domestic product) observed between 2020 and 2030, with a minimum decarbonization rate of 2% per year beyond 2030.

The well-below 2 °C and 1.5 °C scenarios are implemented by limiting end-of-century radiative forcing to 2.6 Wm^−2^ and 1.9 Wm^−2^ (ref. ^65^). Starting in 2025, emissions reductions are pursued cost-effectively across regions via a universal global carbon price on energy-related emissions. This is different from the regionally differentiated approach in the NDC scenario. Cumulative emissions in the well-below 2 °C scenario peak at 900 GtCO_2_ in 2070 and decline to 600 GtCO_2_ in 2100. The 1.5 °C scenario peaks at 420 GtCO_2_ in 2050 and declines below zero by 2100. These limits correspond to cumulative CO_2_ emissions that are below the budgets suggested by the IPCC^1^.

**Fig. 4:**
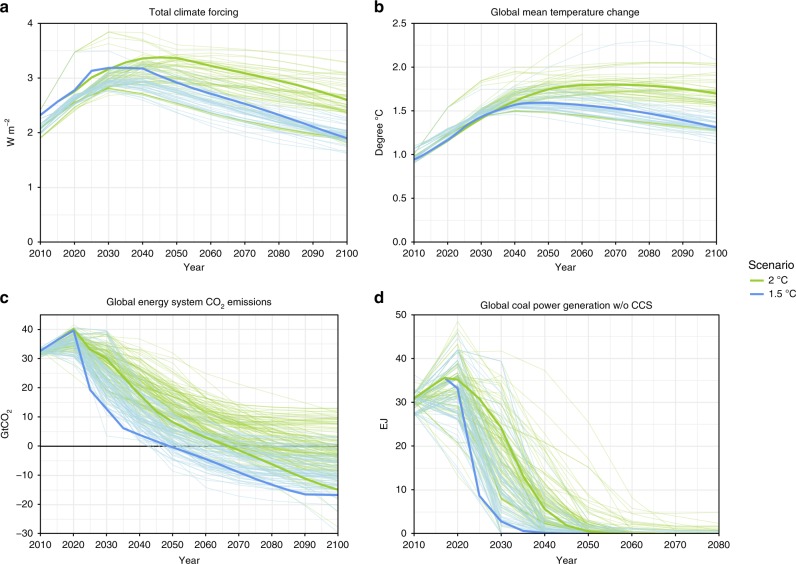
Well-below 2 °C and 1.5 °C scenarios. We compare our scenarios with others in the literature in terms of total climate forcing (**a**), global mean temperature change (**b**), global energy-related CO_2_ emission pathways (**c**), and global coal power generation pathways (**d**). The lighter lines are scenarios from the IAMC 1.5 °C Scenario Explorer^67^. The light blue lines indicate scenarios categorized as below 1.5 °C and 1.5 °C low overshoot, and the light green lines indicate scenarios categorized as lower 2 °C in the database

The use of bioenergy is constrained to a maximum of 200 EJ globally over the simulation period. This limits the maximum level of negative emissions from bioenergy plus carbon capture and storage (BECCS) to approximately −15 GtCO_2_ by 2100 (Fig. 4c). This bioenergy constraint can avoid potentially unrealistic large-scale deployment of negative emissions technologies^66^. Limiting BECCS during the second half of the century induces more aggressive emissions reductions in the near term. Our results are within the range of coal generation pathways in scenarios developed by different integrated assessment models from the IPCC 1.5 °C scenario database^67^ (Fig. 4d) and track recent history more accurately than many of the scenarios.

## Supplementary information

Supplementary Information

Source Data

## Data Availability

The source data underlying Figs. 1 and 2 and Supplementary Figs. 1 and 2 are provided as a Source Data file. All data used for this analysis are available from publicly available sources cited or from the authors upon reasonable request.

## Code availability

Code used in GCAM and R for this work is available from the authors upon reasonable request.

## References

[1] IPCC. Global Warming of 1.5 °C. An IPCC Special Report on the impacts of global warming of 1.5 °C above pre-industrial levels and related global greenhouse gas emission pathways, in the context of strengthening the global response to the threat of climate change, sustainable development, and efforts to eradicate poverty. (Intergovernmental Panel on Climate Change, Geneva, Switzerland, 2018).

[2] UNEP. The Emissions Gap Report 2017. https://wedocs.unep.org/bitstream/handle/20.500.11822/22070/EGR_2017.pdf (United Nations Environment Programme (UNEP), Nairobi, 2017).

[3] Fawcett AA (2015). Can Paris pledges avert severe climate change?. Science.

[4] Peters GP (2017). Key indicators to track current progress and future ambition of the Paris Agreement. Nat. Clim. Change.

[5] Kuramochi T (2018). Ten key short-term sectoral benchmarks to limit warming to 1.5 °C. Clim. Policy.

[6] Iyer G (2017). Measuring progress from nationally determined contributions to mid-century strategies. Nat. Clim. Change.

[7] van Breevoort, P. et al. The Coal Gap: planned coal-fired power plants inconsistent with 2 °C and threaten achievement of INDCs. https://climateanalytics.org/media/cat_coal_gap_briefing_cop21.pdf (Climate Action Tracker, 2015).

[8] Shearer C, Fofrich R, Davis SJ (2017). Future CO_2_ emissions and electricity generation from proposed coal-fired power plants in India. Earth’s Future.

[9] Edenhofer O, Steckel JC, Jakob M, Bertram C (2018). Reports of coal’s terminal decline may be exaggerated. Environ. Res. Lett..

[10] Pfeiffer A, Hepburn C, Vogt-Schilb A, Caldecott B (2018). Committed emissions from existing and planned power plants and asset stranding required to meet the Paris Agreement. Environ. Res. Lett..

[11] Rocha, M. et al. Implications of the Paris Agreement for coal use in the power sector. https://climateanalytics.org/media/climateanalytics-coalreport_nov2016_1.pdf (Climate Analytics, 2016).

[12] Bertram C (2015). Carbon lock-in through capital stock inertia associated with weak near-term climate policies. Technol. Forecast. Soc. Change.

[13] Smith CJ (2019). Current fossil fuel infrastructure does not yet commit us to 1.5 °C warming. Nat. Commun..

[14] Pfeiffer A, Millar R, Hepburn C, Beinhocker E (2016). The ‘2 °C capital stock’ for electricity generation: Committed cumulative emissions from the electricity generation sector and the transition to a green economy. Appl. Energy.

[15] McGlade C, Ekins P (2015). The geographical distribution of fossil fuels unused when limiting global warming to 2C. Nature.

[16] Oberschelp S, Pfister C, Raptis E, Hellweg. S (2019). Global emission hotspots of coal power generation. Nat. Sustain..

[17] Tong D (2018). Targeted emission reductions from global super-polluting power plant units. Nat. Sustain..

[18] Carbon Tracker. Powering down coal: navigating the economic and financial risks in the last years of coal power. https://www.carbontracker.org/wp-content/uploads/2018/12/CTI_Powering_Down_Coal_Report_Nov_2018_4-4.pdf (2018).

[19] Benn, A., Bodnar, P., James Mitchell, J. & Waller, J. Managing the Coal Capital Transition. *Rocky Mountain Institute*. http://www.rmi.org/insight/managing-coal-capital-transition (2018).

[20] IEA. World Energy Model, Sustainable Development Scenario. https://www.iea.org/weo/weomodel (2018).

[21] Davis SJ, Caldeira K, Matthews HD (2010). Future CO_2_ emissions and climate change from existing energy infrastructure. Science.

[22] Davis SJ, Socolow RH (2014). Commitment accounting of CO_2_ emissions. Environ. Res. Lett..

[23] IEA. Tracking Clean Energy Progress 2017. https://www.iea.org/publications/freepublications/publication/TrackingCleanEnergyProgress2017.pdf (2017).

[24] Wilson IAG, Staffell I (2018). Rapid fuel switching from coal to natural gas through effective carbon pricing. Nat. Energy.

[25] China Electric Council. Basic Electric Power Statistics 2017. http://www.cec.org.cn/guihuayutongji/tongjxinxi/niandushuju/2018-12-19/187486.html (2017).

[26] U.S. Energy Information Administration (EIA). Existing capacity by energy source. https://www.eia.gov/electricity/annual/html/epa_04_03.html (2017).

[27] Central Electricity Authority, Government of India, Ministry of Power. Indian installed capacity (MW) of power stations. http://cea.nic.in/reports/monthly/installedcapacity/2017/installed_capacity-12.pdf (2017)

[28] Central Electricity Authority, Government of India, Ministry of Power. National electricity plan. http://www.cea.nic.in/reports/committee/nep/nep_jan_2018.pdf (2018)

[29] Global Energy Monitor, Global Coal Plant Tracker, July 2018, https://endcoal.org/global-coal-plant-tracker/ (2018).

[30] Rogelj J (2016). Paris Agreement climate proposals need a boost to keep warming well below 2C. Nature.

[31] Peters GP, Andrew RM, Solomon S, Friedlingstein P (2015). Measuring a fair and ambitious climate agreement using cumulative emissions. Environ. Res. Lett..

[32] Mills AD, Wiser RH, Seel J (2017). Power Plant Retirements: Trends and Possible Drivers (No. LBNL-2001083)..

[33] Tong D (2018). Current emissions and future mitigation pathways of coal-fired power plants in China from 2010 to 2030. Environ. Sci. Technol..

[34] Celebi, M., Graves, F., Bathla, G., & Bressan, L. Potential coal plant retirements under emerging environmental regulations. *The Brattle Group* **8** (2010).

[35] Fleischman L, Cleetus R, Deyette J, Clemmer S, Frenkel S (2013). Ripe for retirement: an economic analysis of the US coal fleet. Electr. J..

[36] Cornot-Gandolphe, S. Indonesia’s electricity demand and the coal sector: export or meet domestic demand? OIES paper: CL 5. *Oxford Institute for Energy Studies*. 10.26889/9781784670795 (2017).

[37] World Resources Institute. 5 Issues to Watch as India Reaches for Ambitious Energy Access Target, http://www.wri.org/blog/2017/02/5-issues-watch-india-reaches-ambitious-energy-access-target (2017).

[38] Dobrotkova Z, Surana K, Audinet P (2018). The price of solar energy: comparing competitive auctions for utility-scale solar PV in developing countries. Energy Policy.

[39] Rubin, E. S., Davison, J. E. & Herzog, H. J. The cost of CO2 capture and storage. *Int. J. Greenh. Gas Control* **40**, 378–400 (2015).

[40] U.S. EIA. Levelized cost and levelized avoided cost of new generation resources in the annual energy outlook 2019, https://www.eia.gov/outlooks/aeo/pdf/electricity_generation.pdf (2019).

[41] Smith P (2015). Biophysical and economic limits to negative CO2 emissions. Nat. Clim. Change.

[42] Europe Beyond Coal. Overview: National coal phase-out announcements in Europe. https://beyond-coal.eu/wp-content/uploads/2017/12/National-phase-out-overview-171219.pdf. (2017).

[43] Government of Canada, Powering Past Coal Alliance Declaration. https://www.canada.ca/en/services/environment/weather/climatechange/canada-international-action/coal-phase-out/alliance-declaration.html (2019).

[44] Ministry of Trade, Industry and Energy. Ministry Announces 8th Basic Plan for Electricity Supply and Demand. Republic of Korea. http://english.motie.go.kr/en/tp/energy/bbs/bbsView.do?bbs_seq_n=605&bbs_cd_n=2&view_type_v=TOPIC&&currentPage=1&search_key_n=&search_val_v=&cate_n=3 (2017).

[45] National Development and Reform Committee of the People’s Republic of China. 2017 List of halted and delayed coal construction projects by province. http://www.escn.com.cn/news/show-465553.html (2017).

[46] Bjarne Steffen, Tobias S Schmit (2019). A quantitative analysis of 10 multilateral development banks’ investment in conventional and renewable power-generation technologies from 2006 to 2015. Nat. Energy.

[47] Burchart-Korol D, Fugiel A, Czaplicka-Kolarz K, Turek M (2016). Model of environmental life cycle assessment for coal mining operations. Sci. Total Environ..

[48] Zhang, Y. -L. & Cao, F. Fine particulate matter (PM2.5) in China at a city level. *Sci. Rep.* **5**, 14884 (2015). 10.1038/srep14884 PMC4606739 26469995

[49] Keep it in the ground. End New Fossil Fuel Development, http://keepitintheground.org/#read-the-letter (2019).

[50] Bloomberg Philanthropies. Moving America Beyond Coal, https://beyondcoal.bloomberg.org/ (2019).

[51] Green F (2018). Anti-fossil fuel forms. Clim. Change.

[52] Grant Wilson, I. A. & Staffell, I. Rapid fuel switching from coal to natural gas through effective carbon pricing. *Nat. Energy* **3**, 365–372 (2018).

[53] The State Council of the People’s Republic of China. Three-year plan of action for winning the war to protect blue skies. http://www.gov.cn/zhengce/content/2018-07/03/content_5303158.htm (2018).

[54] Shearer, C., Yu, A. & Nace, T. Tsunami warning: can China’s Central Authorities stop a massive surge in new coal plants caused by provincial overpermitting? https://endcoal.org/wp-content/uploads/2018/09/TsunamiWarningEnglish.pdf (2018).

[55] IPCC. Climate Change 2014: Mitigation of Climate Change. Contribution of Working Group III to the Fifth Assessment Report of the Intergovernmental Panel on Climate Change (Intergovernmental Panel on Climate Change, Cambridge, United Kingdom, and New York, NY, USA, 2014).

[56] IPCC. Contribution of Working Group III to the Fourth Assessment Report of the Intergovernmental Panel on Climate Change. (Intergovernmental Panel on Climate Change, 2007).

[57] IPCC. Contribution of Working Group III to the Third Assessment Report of the Intergovernmental Panel on Climate Change (Intergovernmental Panel on Climate Change, 2001).

[58] Thomson AM (2011). RCP4.5: a pathway for stabilization of radiative forcing by 2100. Clim. Change.

[59] Calvin K (2017). “The SSP4: A world of deepening inequality.”. Glob. Environ. Change.

[60] Hartin CA, Patel P, Schwarber A, Link RP, Bond-Lamberty BP (2015). A simple object-oriented and open-source model for scientific and policy analyses of the global climate system – Hectorv1.0. Geosci. Model Dev..

[61] Riahi (2017). The Shared Socioeconomic Pathways and their energy, land use, and greenhouse gas emissions implications: an overview. Glob. Environ. Change.

[62] IEA. World Energy Statistics and Balances 2018, 10.1787/25186442 (2018).

[63] BJX news. 2018 Coal power phase-out target by province (in Chinese), http://news.bjx.com.cn/html/20180508/896297.shtml (2018).

[64] Sierra club. Coal pollution in America, https://content.sierraclub.org/coal/coal-plant-map (2019)

[65] Rogelj J (2018). Scenarios towards limiting global mean temperature increase below 1.5 °C. Nat. Clim. Change.

[66] Smith (2015). Biophysical and economic limits to negative CO2 emissions. Nat. Clim. Change.

[67] IIASA. IAMC 1.5 °C Scenario Explorer. https://data.ene.iiasa.ac.at/iamc-1.5c-explorer/ (2018).

