# Supplementary material for: Quantifying operational lifetimes for coal power plants under the Paris goals
Source: Nat Commun. 2019 Oct 18;10:4759. doi: 10.1038/s41467-019-12618-3 (PMC6800419; doi:10.1038/s41467-019-12618-3)
Supplement: Supplementary file 1 — Supplementary Information [file 41467_2019_12618_MOESM1_ESM.pdf]

Quantifying operational lifetimes for coal power plants under the Paris goals

Cui et al.

Supplementary Information

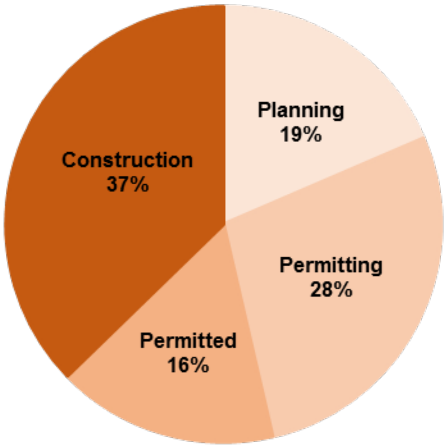

Supplementary Figure 1 Global proposed coal power capacity by development stage.

[Source data are provided as a Source Data file.]

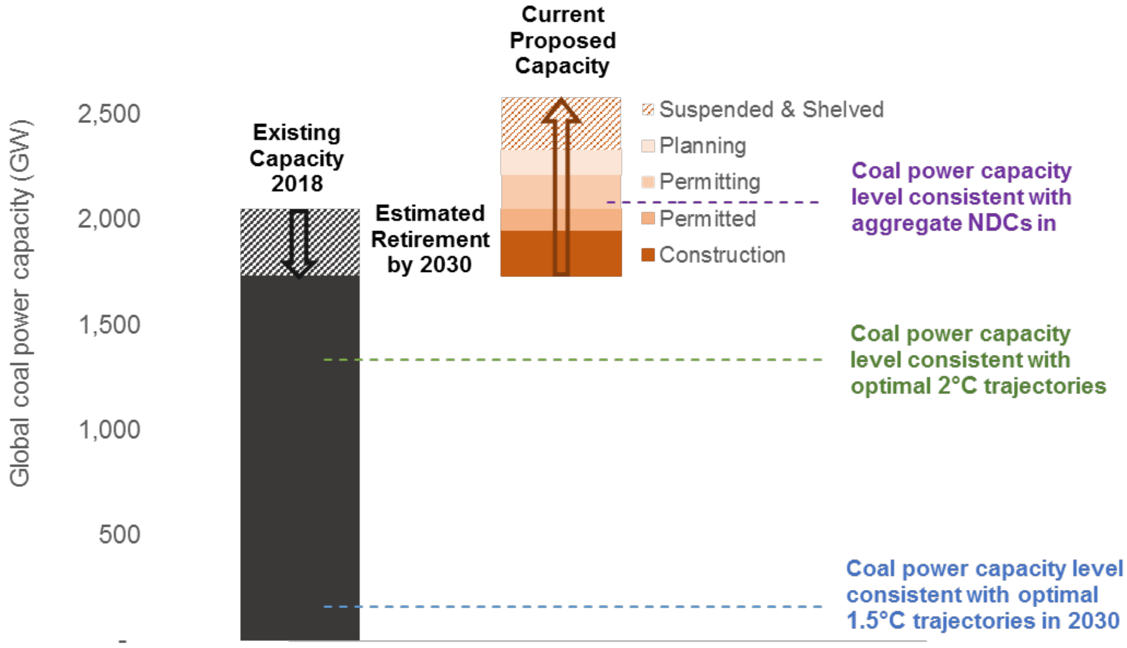

Supplementary Figure 2 Global coal power capacity in 2030 compared to levels consistent with near- and long-term climate goals.

[Source data are provided as a Source Data file.]

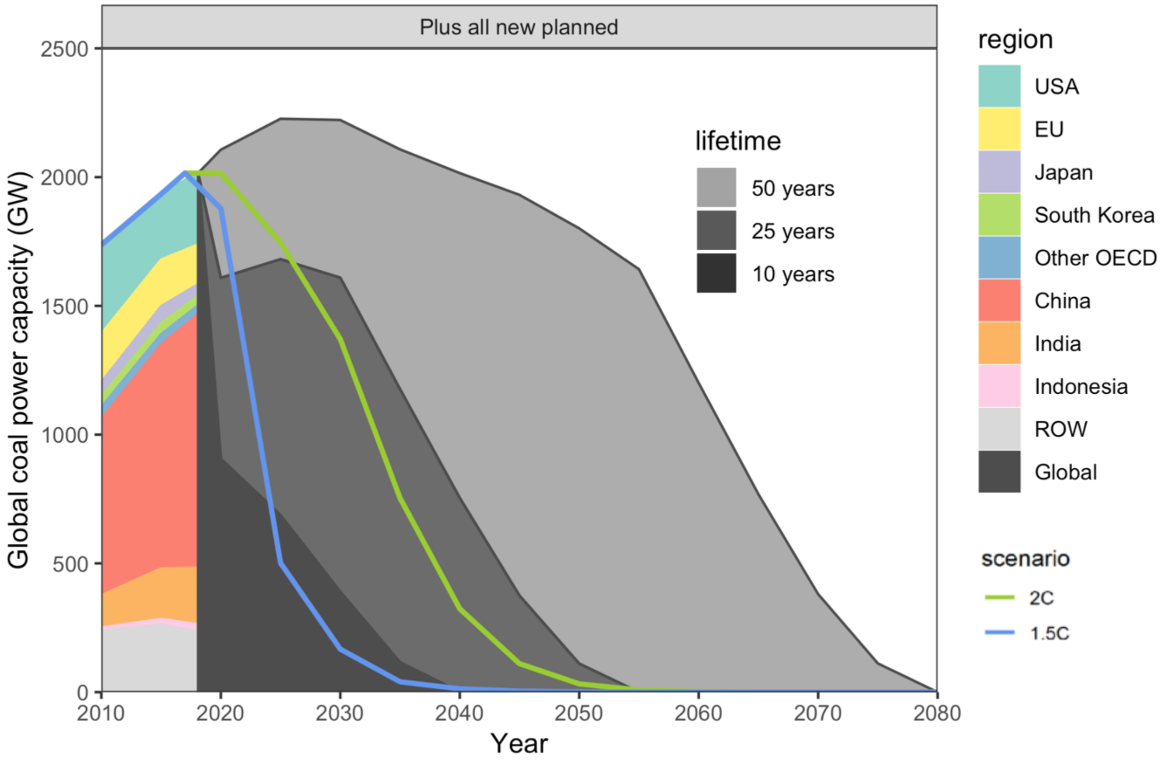

Supplementary Figure 3 Lifetime limits for coal power plants if proposed plants in all phases come online. We show global coal power capacity under different plants lifetimes compared to capacity levels consistent with a 2°C (green) and 1.5°C (blue) pathway, for a case where all proposed plants come online as scheduled, including those currently under construction, as well as in planning or permitting stages.

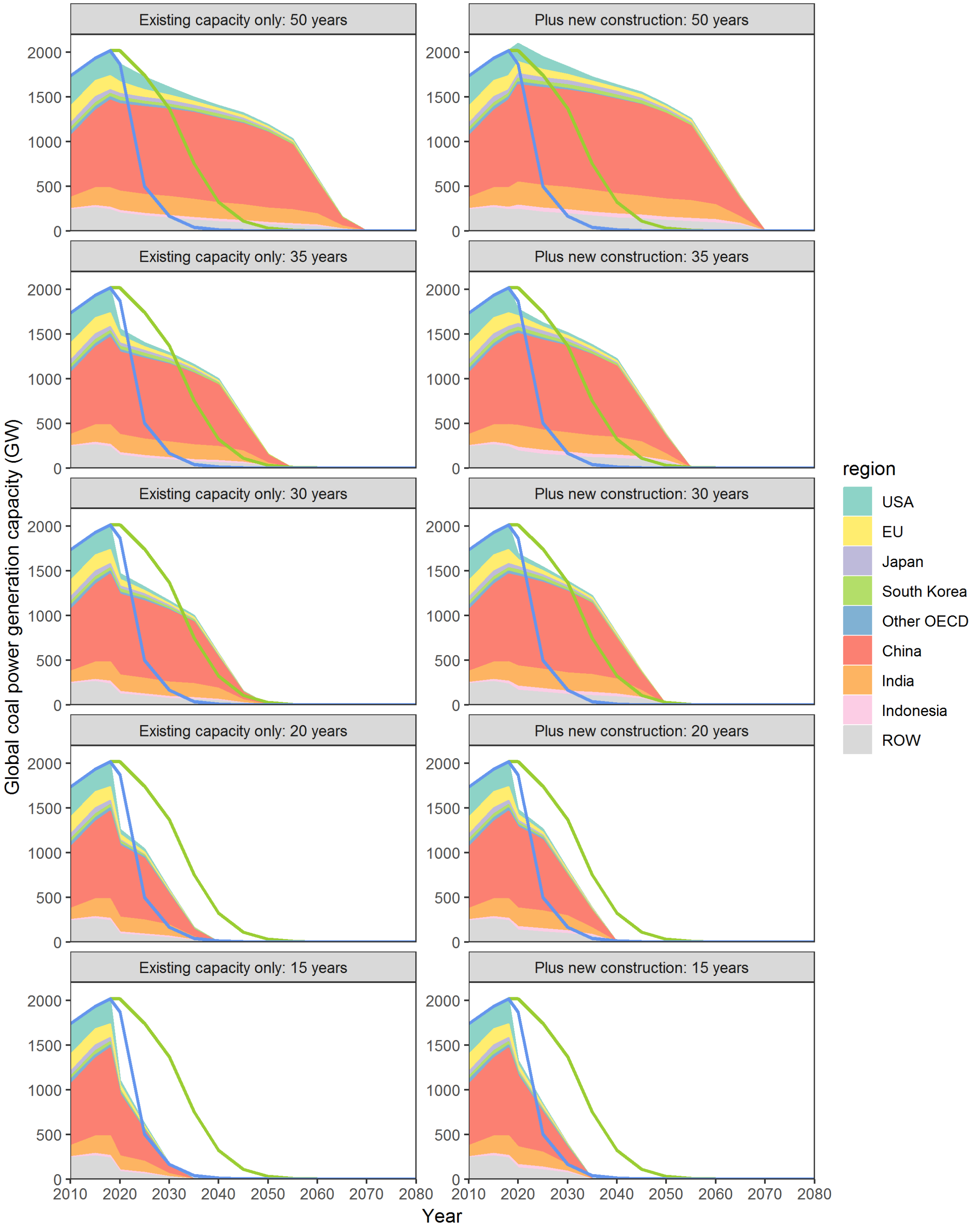

Supplementary Figure 4 Regional coal capacity pathways under different lifetime limits. We examine coal power capacity by region under different plant lifetimes, compared to capacity levels consistent with a 2°C (green) and 1.5°C (blue) pathway, for a case where no new coal plants are built and where plants currently under construction come online as scheduled, but those in planning or permitting stages are not built.

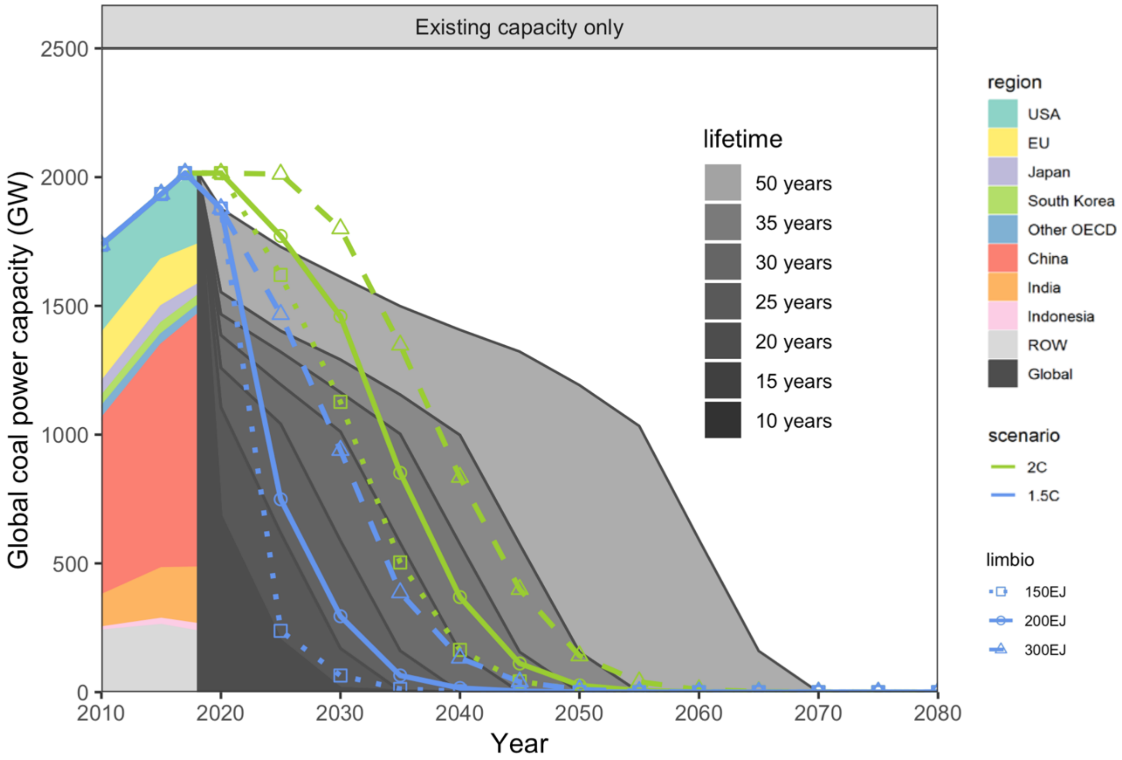

Supplementary Figure 5 Lifetime limits for coal power plants under lower bioenergy limits. We show global coal power capacity under different operational lifetime limits, compared to capacity levels consistent with a 2°C (green) and 1.5°C (blue) pathway, under different levels of bioenergy availability and for the case where no new capacity comes online.

Supplementary Table 1 Capacity factors of coal power plants by GCAM region

GCAM region name	Capacity factor [1]
Africa_Eastern	0.73
Africa_Northern	0.73
Africa_Southern	0.73
Africa_Western	0.73
Argentina	0.43
Australia_NZ	0.65
Brazil	0.44
Canada	0.62
Central America and Caribbean	0.43
Central Asia	0.62
China	0.54
Colombia	0.43
EU-12	0.51
EU-15	0.51
Europe_Eastern	0.41
Europe_Non_EU	0.41
European Free Trade Association	0.51
India	0.60
Indonesia	0.62
Japan	0.62
Mexico	0.64
Middle East	0.21
Pakistan	0.62
Russia	0.38
South Africa	0.73
South America_Northern	0.43
South America_Southern	0.43
South Asia	0.62
South Korea	0.82
Southeast Asia	0.62
Taiwan	0.62
USA	0.66

[1] U.S. Energy Information Administration. Electric generator capacity factors vary widely across the world. September 8, 2015. https://www.eia.gov/todayinenergy/detail.php?id=22832

Supplementary Table 2 GCAM efficiency assumptions for conventional coal generation by vintage group

Vintage year	Efficiency
2010	0.397
2015	0.397
2020	0.41
2025	0.422
2030	0.434
